# Living alone and mortality among older people in Västerbotten County in Sweden: a survey and register-based longitudinal study

**DOI:** 10.1186/s12877-019-1330-9

**Published:** 2020-01-06

**Authors:** Nawi Ng, Ailiana Santosa, Lars Weinehall, Gunnar Malmberg

**Affiliations:** 1 Department of Epidemiology and Global Health, Faculty of Medicine, Umeå University, Umeå, Sweden; 2 Centre for Demographic and Ageing Research, Umeå University, Umeå, Sweden; 3 Global Health, School of Public Health and Community Medicine, Institute of Medicine, Sahlgrenska Academy, University of Gothenburg, Gothenburg, Sweden; 4 Department of Geography, Umeå University, Umeå, Sweden

**Keywords:** Living alone, Older people, Social support, Family network, Living arrangement, Deaths

## Abstract

**Background:**

Living alone is increasingly common and has been depicted as an important cause of mortality. We examined the association between living alone and mortality risks among older men and women in northern Sweden, by linking two unique longitudinal datasets.

**Methods:**

We used the Linnaeus database, which links several population registers on socioeconomic and health. This register-based study included 22,226 men and 23,390 women aged 50 and 60 years in Västerbotten County who had participated in the Västerbotten Intervention Program (VIP) during 1990–2006, with a total of 445,823 person-years of observation. We conducted Cox-proportional hazard regression to assess the risk of living alone on the mortality that was observed between 1990 and 2015, controlling for socio-demographic factors, chronic disease risk factors and access to social capital.

**Results:**

Older men and women who lived alone with no children at home were at a significantly higher risk of death compared to married/cohabiting couples with children at home (with an adjusted hazard ratio of 1.38, 95% CI of 1.26–1.50 in men and 1.27, 95% CI of 1.13–1.42 in women). Living alone was an even stronger factor than the well-established chronic disease risk factors and a lack of access to social capital.

**Conclusions:**

A significant association between living alone and mortality among the older adult population in Sweden was observed. Providing good social support for older people is important in preventing the negative health impact of living alone.

## Introduction

Living alone is becoming increasingly common in high-income countries, due to for instance trends of longevity, high divorce rates, low rates of intergenerational co-residence, and high rates of widowhood [1–3]. The trends are more advanced in some countries, where Sweden reports the highest prevalence of living alone in Europe with more than 50% of the households being single household [4]. At the same time, it has been claimed that living alone is a major risk factor for mortality and morbidity not least for the older people for several reasons [5–7]. It may cause social isolation, and if not compensated by other forms of social capital can result in feeling of loneliness and depression with negative consequences on health and survival [8, 9]. The effect of social isolation on mortality is comparable to traditional clinical risk factors [7, 10]. People in single households maybe more prone to adopt poor health behaviours [9, 11]. Living alone is also more costly and may add to the economic burdens of people with low socio-economic position. In addition, unemployment and retirement may lead to social isolation with more serious consequences on health and mortality for those living alone [12]. Moreover, people living alone are more likely to experience unmet care needs [13].

In this study, we examine the association between living alone and mortality among men and women aged 50 and 60 years in Västerbotten County. This study provides a novel contribution to the literature by linking data from the comprehensive data of the Västerbotten Intervention Programme (VIP) with Swedish national register data that allow a longitudinal follow-up of survival among the Swedish older population. By including various covariates in the analysis, we have explored the association between living alone and mortality after adjusting for lifestyle factors, socioeconomic position, ill-health and access to social capital. Being a forerunner in the extent of living alone, the Swedish case is special but interesting since it may inform us about the coming trends in other European countries and elsewhere.

## Methods

### Study design and population

We used the longitudinal information from the Linnaeus database, which links the Swedish population register, socio-economic data, hospitalisation and the Swedish death register at national level with the comprehensive VIP data (see below) during the years 1990–2006. Details about the Linnaeus database have been presented elsewhere [14].

In this study, we analysed the population in Västerbotten County who participated in the VIP. In brief, the VIP is a combined individual- and population-based health intervention programme for reducing the burden of cardiovascular disease risk factors. Individuals who turn 40, 50, and 60 years old in the county were invited for health screening which included questionnaire, anthropometric and blood pressure measurement, and blood sampling. Trained nurses provided feedback to each individual in a counselling session [15]. In this paper, we focused on individuals aged 50 and 60 years who had participated for the first time in the VIP during the years 1990–2006 (*n* = 45,616, with a total follow-up time of 445,823 person-years). As the VIP data is linked to the population register data in the Linnaeus database, we obtained their socioeconomic and living arrangement data in the same year as when the individuals participated in the VIP examination, as well as death date for those who died.

### Outcome variables

We recorded deaths occurring among the study participants until October 2015.

### Independent variables

The main independent variable of interest was living alone, constructed from family type and living arrangement data. We categorised the ‘living alone’ variable into four categories of (i) married or cohabited with children living at home, (ii) married or cohabited without children living at home, (iii) single parent with children living at home, and (iv) single parent without children living at home. We defined the participants as ‘living alone’ if they belonged to the last category.

### Sociodemographic variables

Socio-demographic variables included were: sex (men and women); age group (50, 60 years); and education level (basic, middle, and high education). The employment variable was categorised into full-time employment, intermittent employment, mostly unemployed, fully unemployed, partly retired, and fully retired.

### Chronic disease risk factor variables

Smoking status and smokeless tobacco (referred to as snus in Sweden) users were assessed based on the question “Do you currently smoke?” and “Do you currently use snus?”. We dichotomised the responses into ‘never/non-current’ and ‘current smokers/snus users’. Individuals who responded positively to at least two of the CAGE questions were categorised as having a risk of alcohol dependency. CAGE is the acronym of the four questions in the CAGE questionnaire, which measures if the respondents: had felt the need to **C**ut down on drinking, had been **A**nnoyed by people who criticised the drinking, had ever felt **G**uilt about drinking, and had felt the need to drink first thing in the morning as **E**yeopener [16]. Physical activity was measured using questions on commuting, recreational time, and physical exercise. Responses from these physical activity questions were analysed and later classified into ‘sedentary’, ‘moderate’ or ‘physically active’. Details about the construction of the physical activity variable have been published elsewhere [17]. Body mass index (BMI) was categorised into ‘under/normal weight’ (BMI < 25 kg/m^2^) and ‘overweight or obese’ (BMI ≥ 25 kg/m^2^). Respondents were categorised as diabetic if they had fasting blood glucose of ≥7 mmol/L or 2-h blood glucose of ≥12.2 mmol/L. Cholesterol level was dichotomised into ‘normal’ or ‘hypercholesterolemia’ (if blood cholesterol ≥6.5 mmol/L). Respondents were classified as having high blood pressure if the average systolic blood pressure was ≥140 mmHg or the diastolic blood pressure was ≥90 mmHg, or if they had been taking blood pressure-lowering medication in the last 14 days.

### Social capital variables

Social capital was measured based on self-reported information on social participation and informal socialising. The respondents were asked if they participated in any leisure-time activity or volunteer organisation during the last year, and if so, how often. They were also asked the number of people: (i) who they know and interact with; (ii) who can come to their house anytime and feel at home; and (iii) who were family or friends with whom they can speak their mind openly. Based on their responses, participants were categorised into having ‘low’, ‘medium’ and ‘high’ levels of social participation and informal socialising. The details on how social capital was constructed has been published earlier [18].

### Statistical analyses

As many of the chronic disease risk factors are related to each other, the inclusion of the factors as separate variables in a multivariable regression analysis will cause the problem of multicollinearity. We, therefore, performed a multivariate analysis to reduce the dimension of the risk factor data. We created an index of chronic disease risk factors using factor analysis with oblique rotation and three-factor solution allowing for polychoric correlation between the variables (Additional file 1: Table S1) for men and women separately. Based on the indices, participants were arbitrarily categorised with either a low load of risk factors (the lowest 60%) or with a high load of risk factors (the top 40%). About 29.6% of 45,616 individuals in this study had missing data in at least one of the variables used in the analysis, mainly from the alcohol dependency variable (22.8% missing values), the social participation variable (4.5%), the smokeless tobacco use variable (4.2%) and the diabetes variable (4%). We therefore conducted multiple imputations – with a total of ten imputations – based on Rubin’s approach for the imputation of missing data [19].

We presented descriptive statistics as to the socio-economic and social capital indicators of the study participants. We conducted Cox-proportional hazard regression analysis to analyse the hazard ratio of living alone among adults on their subsequent mortality, controlling for socio-demographic variables, chronic disease risk factor load and social capital variables. We tested for the interaction between living arrangement and social capital variables and found no significant interaction (data not shown).

As a sensitivity analysis, we also conducted a similar analysis for all population age 50 and over who lived in the Västerbotten County in 1990 and followed them until death or censored in October 2015. For this analysis, we did not control for chronic disease risk factors and access to social capital as this information were not available in the register data. All analyses were conducted in Stata 15 (Stata Corp Texas, 2017).

## Results

We analysed a total of 45,616 individuals aged 50 and 60 years who had first participated in the VIP during the years 1990–2006, with a total of 7019 deaths observed within 445,823 person-years of observation. These yielded an overall mortality rate of 19.1 per 1000 person-years in men and 12.7 in women (Table 1). As the mortality rates differed between those with and without missing data (Additional file 1: Table S2), we used the multiplied imputed data for subsequent analyses.

**Table 1:** Mortality rate in the VIP study population

Variables	VIP participants		
	50 (*n* = 25,441)	60 (*n* = 20,175)	Total (*n* = 45,616)
Men			
Number population	12,450	9776	22,226
Number of deaths (up to Oct 31, 2015)	1292	2765	4057
Total follow-up (year)	119,657	92,800	212,457
Mortality rate per 1000 person-year	10.8	29.8	19.1
Women			
Number population	12,991	10,399	23,390
Number of deaths (up to Oct 31, 2015)	909	2053	2962
Total follow-up (year)	129,012	104,354	233,366
Mortality rate per 1000 person-year	7.1	19.7	12.7

The characteristics of the study respondents are summarised in Table 2. About 19% of men and 24% of women had a high education. We observed a high proportion of the individuals in the VIP cohort who lived in partnership with children at home (40% among men and 30% among women) and were fully employed (about 72% in men and women). About 22% men and 21% women reported living in a single-family household with no children at home. About 40% of men and 44% of women in the VIP study reported having no access to social participation, while in contrast, 71% of men and women reported having high access to the informal socialising form of social capital.

**Table 2:** Socio-demographic characteristics and social capital levels in the VIP participants

Variables	VIP participants aged 50 and 60	
	Men (*n* = 22,226) % (95% CI)	Women (*n* = 23,390) % (95% CI)
Family type and living arrangement (%)		
In partnership with children at home	39.3 (38.7–40.0)	29.9 (29.3–30.5)
In partnership without children at home	34.8 (34.2–35.4)	42.8 (42.1–43.4)
Single parent with children at home	4.0 (3.8–4.3)	6.7 (6.4–7.0)
Single with no children at home	21.8 (21.3–22.4)	20.6 (20.1–21.2)
Employment status (%)		
Fully employed	72.1 (71.5–72.6)	72.2 (71.6–72.8)
Employed with unemployed	5.5 (5.2–5.8)	5.8 (5.5–6.1)
Mostly unemployed	3.4 (3.2–3.7)	2.0 (1.8–2.1)
Fully unemployed	4.0 (3.8–4.3)	3.4 (3.2–3.6)
Partly pensioner	5.0 (4.7–5.3)	4.9 (4.6–5.1)
Fully pensioner	9.9 (9.5–10.3)	11.7 (11.3–12.1)
Education level (%)		
Basic education	33.4 (32.8–34.0)	27.2 (26.7–27.8)
Middle education	47.9 (47.3–48.6)	48.6 (48.0–49.3)
High education	18.7 (18.2–19.2)	24.2 (23.6–24.7)
Social participation (%)		
High access	23.8 (23.2–24.4)	26.0 (25.5–26.6)
Medium access	23.1 (22.6–23.7)	20.3 (19.8–20.8)
Low access	13.1 (12.6–13.5)	9.9 (9.5–10.3)
No access	40.0 (39.4–40.7)	43.8 (43.1–44.5)
Informal socializing (%)		
High access	70.6 (70.0–71.2)	71.3 (70.7–71.9)
Medium access	17.7 (17.2–18.2)	17.1 (16.6–17.6)
Low access	8.0 (7.6–8.3)	7.6 (7.3–8.0)
No access	3.8 (3.5–4.0)	3.9 (3.7–4.2)

In addition to the variables above, we also had information about risk factor quintiles for individuals who participated in the VIP program during 1990–2006. As the risk factor quintiles were approximately equally distributed; hence we do not present the information in this table. The proportions and 95% confidence intervals for the VIP participants are based on imputed data using the Rubin approach. See text in the Methods section for more details

In the Cox regression analysis (Table 3), we observed that those who lived alone were at a much higher risk of death compared to those who were in a partnership and with children at home (hazard ratio/HR = 1.38, 95% CI = 1.26–1.50 in men and HR = 1.27, 95% CI = 1.13–1.42 in women), after controlling for other sociodemographic variables, as well as social capital and risk factor burden. Men who were single parents with children at home also exhibited an approximately similar level of risk of mortality (HR = 1.26, 95% CI = 1.06–1.49). We observed that men without full employment, or those who were pensioners, had a significantly higher risk of mortality when compared to their counterparts who had full employment. Among women, the effects were significant for pensioners and those with intermittent employment. A significant association between no/low access to social participation and mortality was observed in both men (HR = 1.12, 95% CI = 1.04–1.20) and women (HR = 1.10, 95% CI = 1.01–1.19), but not observed for a lack of informal socialising after adjustments were made for other factors.

**Table 3:** Hazard ratio of mortality related to living alone among the VIP participants

Variables	VIP participants aged 50 and 60 recruited during 1990–2006	
	Men (*n* = 22,226) Hazard Ratio (95% Confidence Interval)	Women (*n* = 23,390) Hazard Ratio (95% Confidence Interval)
Age group		
50 years	Reference	Reference
60 years	**1.84 (1.70–1.98)**	**1.66 (1.52–1.82)**
Family type and living arrangement		
In partnership with children at home	Reference	Reference
In partnership without children at home	1.08 (0.99–1.17)	1.04 (0.94–1.15)
Single parent with children at home	**1.26 (1.06–1.49)**	0.95 (0.79–1.14)
Single with no children at home	**1.38 (1.26–1.50)**	**1.27 (1.13–1.42)**
Employment status		
Fully employed	Reference	Reference
Intermittent employment	**1.29 (1.12–1.49)**	1.06 (0.88–1.26)
Mostly unemployed	**1.36 (1.16–1.61)**	**1.61 (1.22–2.12)**
Fully unemployed	**1.35 (1.15–1.58)**	1.12 (0.93–1.35)
Partly pensioner	**1.14 (1.01–1.28)**	**1.48 (1.28–1.70)**
Fully pensioner	**1.58 (1.45–1.72)**	**1.53 (1.39–1.68)**
Education level		
High education	Reference	Reference
Middle education	1.11 (1.00–1.23)	1.11 (0.98–1.24)
Basic education	1.05 (0.94–1.17)	1.06 (0.93–1.19)
1st risk factor load		
Low risk load (60% lowest)	Reference	Reference
High risk load (40% highest)	1.02 (0.93–1.11)	1.01 (0.89–1.15)
2nd risk factor load		
Low risk load (60% lowest)	Reference	Reference
High risk load (40% highest)	**1.11 (1.03–1.21)**	1.09 (0.93–1.27)
3rd risk factor load		
Low risk load (60% lowest)	Reference	Reference
High risk load (40% highest)	1.08 (1.00–1.17)	**1.20 (1.08–1.32)**
Social participation		
High / medium access	Reference	Reference
Low / No access	**1.12 (1.04–1.20)**	**1.10 (1.01–1.19)**
Informal socializing		
High / medium access	Reference	Reference
Low / No access	1.05 (0.95–1.17)	1.05 (0.94–1.17)

Only 95% CIs which have significance are bolded.

The analysis on the whole Västerbotten population age 50 and over also showed a similar higher risk of mortality among individuals who lived alone with a hazard ratio of 1.32 (95% CI = 1.26–1.37) among men and 1.20 (95% CI = 1.14–1.26) among women (Additional file 1: Table S3), which were quite similar to the hazard ratio observed among the VIP participants when also controlled chronic disease risk factors and access to social capital.

## Discussions

In this study, we found an association between living alone and mortality among the older population in Västerbotten County. This phenomenon could be an important public health issue in light of the ageing transition and how society and its health care systems adapt to it. Additional analyses using the Linnaeus database indicated an increase in the proportion of adults aged 40+ who lived alone in the Västerbotten County, from 29% in 1990 to 40% in 2006. During the same period, the proportion of divorcees and widow/widower adults aged 40+ increased. In 2006, a majority of older people aged 60–69 years in Västerbotten County reported living alone (data not shown). Similar trends were observed in the US [2, 20]. More US men aged 65+ preferred to live with their spouse, in contrast with women in a similar age group. Along with the changes in living arrangements among the US population, the network size decreased, and social isolation increased during 1985–2004 [20]. Ruggles reported a substantial decline in the proportion of older people aged 65+ who resided with their children age 18+ between 1850 and 1990 (54% among whites and 28% among blacks) and concluded that changes in living arrangements were due to the increased opportunities for the younger generation in the last century [2].

The causal pathways of living alone and mortality are multi-faceted. People who lived alone tended to have a more restricted social network (social isolation), poorer health, and a higher risk of mortality. In the present study, we found that the unemployed and pensioners had a higher mortality risk compared to other population groups. Beyond retirement age, older people’s families and social networks tend to shrink. An increasing trend towards the formation of smaller offspring nuclear families, the progression towards widowhood, worsening health conditions, limitations on physical mobility, and natural selection of mortality among peers, exposed older people to the threat of physical and social isolation. Our findings strengthen the evidence that the association between living alone and mortality persisted significantly, even after controlling for social participation and informal socialising. Social participation was consistently significant in its association with mortality for both men and women, but not with regards to informal socializing.

Using the English Longitudinal Study of Ageing, Shankar et al. showed that older people who experienced social isolation and loneliness, especially those with a lower education, had poorer cognitive function within a four-year follow-up period [21]. Steptoe et al. used the same dataset and observed that social isolation was associated with a higher risk of mortality, but not due to the emotional experience of loneliness. Though both social isolation and loneliness were associated with a poorer quality of life, unlike social isolation, the effect of loneliness was not independent of demographic variables and health problems [22]. A meta-analytic review published in 2010 synthesised findings across 148 studies globally and concluded that people with stronger social relationships have a 50% increased likelihood of survival, and this positive effect is comparable to the negative effect of smoking, obesity, and physical inactivity [7]. Other studies have reported an interaction between a feeling of loneliness and living alone [23, 24], and suggested that the absence of depression and a functional social network are significant predictors of not feeling lonely despite living alone [23]; particularly if social support from friends following widowhood exist [24]. In this study, we observed that the effect of living alone increased among men and women when chronic NCD risk factors and social capital were controlled in the subset analysis.

Our findings provide strong evidence for public and health policy-making that society has to be prepared for the negative outcomes of growing isolation and loneliness when the number of people living alone is increasing. Therefore, special measures to care for and assist lonely older people are necessary through proactive housing policies and the design of neighbourhoods. Theoretical-based interventions, such as those based on self-management of a well-being theory [25] or the formation of social ties [26], especially those ties involving social activity and support within a group format, are more effective in preventing social isolation among older people [27].

### Strengths and limitations of the study

Our study has some strengths, in that we were able to follow-up the VIP population for 26 years and assess the impact of its living arrangements on its mortality, while controlling for several socio-economic, biological risk factor and social capital variables. Multiple imputations were conducted to deal with missing data observed in the VIP study and to reduce the bias. We also acknowledge some unavoidable limitations in this study. Swedish register data only includes information about cohabitants who have common children. This means that the number of non-married cohabitants is underestimated and the number living alone is slightly overestimated. But since this study focuses on the older generation where cohabitation without children and without marriage is rare, this has a marginal effect on the conclusion from our study. Second, we could not assess the effect of the feeling of loneliness on mortality within this study as an independent factor, because the VIP does not measure subjective loneliness. Third, this study did not focus on exploring the pathway between living alone and mortality, for example, if individuals who live alone are more prone to adopt certain lifestyle behaviours, or might have more medical conditions, poorer physical and cognitive functions, which in turn increase their mortality risk. Understanding of the mechanisms of living alone and mortality will allow tailoring intervention strategies to prevent the negative impacts of living alone among the older population.

## Conclusions

Association between living alone and mortality among the adult population aged 50 and 60 years old in Västerbotten County in Sweden was observed and the association persisted even after controlling for chronic disease risk factors and social capital. Therefore, it is important for policy makers to reduce the health and societal impacts of living alone, by focusing on designing supportive housing structures and neighbourhoods that will provide good social support for people who live alone, especially older people, in order to prevent the negative health impacts of living alone.

## Supplementary information

**Additional file 1: Table S1.** Factor loadings of components of chronic disease risk factors among men and women in the VIP. **Table S2.** Mortality rate among VIP participants with complete and incomplete data in the analysis. **Table S3.** Hazard ratio of mortality related to living alone among the Västerbotten population in 1990.

## Data Availability

The Linnaeus database contains anonymised individual data from surveys and Swedish population register data, which is maintained in secure server located at Umeå University and are not publicly available.

## Abbreviations

BMI: Body mass index
HR: Hazard ratio
VIP: Västerbotten Intervention Program
