# Supplementary material for: Living alone and mortality among older people in Västerbotten County in Sweden: a survey and register-based longitudinal study
Source: BMC Geriatr. 2020 Jan 6;20:7. doi: 10.1186/s12877-019-1330-9 (PMC6945693; doi:10.1186/s12877-019-1330-9)
Supplement: Supplementary file 1 — Additional file 1: Table S1. Factor loadings of components of chronic disease risk factors among men and women in the VIP. Table S2. Mortality rate among VIP participants with complete and incomplete data in the analysis. Table S3. Hazard ratio of mortality related to living alone among the Västerbotten population in 1990. [file 12877_2019_1330_MOESM1_ESM.docx]

**Supplementary Tables**

**Living alone and mortality among older people in Västerbotten County in Sweden:**

**a survey and register-based longitudinal study**

Nawi Ng, Ailiana Santosa, Lars Weinehall, Gunnar Malmberg

BMC Geriatrics

**Table S1. Factor loadings of components of chronic disease risk factors among men and women in the VIP**

| **Variables** | **Men** | | | **Women** | | |
| --- | --- | --- | --- | --- | --- | --- |
|  | **Factor 1** | **Factor 2** | **Factor 3** | **Factor 1** | **Factor 2** | **Factor 3** |
| Current smokers | -0.0792 | **0.4816** | 0.0356 | -0.1072 | -0.0686 | **0.5159** |
| Current smokeless tobacco users | -0.0063 | -0.0692 | **0.4042** | -0.0229 | **0.4793** | -0.0693 |
| Physical inactivity | 0.0193 | **0.3386** | 0.1545 | 0.0908 | 0.0703 | **0.3597** |
| Diabetes | **0.5381** | 0.0303 | -0.0011 | **0.5444** | 0.0396 | 0.0140 |
| Hypertension | **0.5961** | 0.0069 | -0.0695 | **0.5644** | -0.1506 | -0.0488 |
| Alcohol dependency | 0.0280 | 0.1067 | **0.3107** | 0.0197 | **0.3435** | 0.1877 |
| Cholesterol level | 0.0340 | 0.1741 | -0.0610 | 0.1332 | -0.2552 | 0.1473 |
| Body mass index | **0.5358** | -0.0494 | 0.0852 | **0.4669** | 0.0185 | -0.0179 |

Table S1 presents the factor loadings of components based on chronic disease risk factors analysed using factor analysis with oblique rotation and three-factor solution allowing for polychoric correlation between the variables. Factor loadings above 0.3 are bolded.

**Table S2. Mortality rate among VIP participants with complete and incomplete data in the analysis**

| **Variables** | | **Age when participating in the VIP program** | |
| --- | --- | --- | --- |
|  |  | **50**  **(n=25 441)** | **60**  **(n=20 175)** |
| **Respondents with complete data in all variables included in this study** | | | |
| **Men** | |  |  |
|  | Number population | 9 260 | 6 385 |
|  | Number of deaths | 906 | 1599 |
|  | Total follow-up (year) | 84 528 | 55 937 |
|  | Mortality rate per 1000 person-year | 10.72 | 28.59 |
| **Women** | |  |  |
|  | Number population | 8 810 | 5 583 |
|  | Number of deaths | 566 | 911 |
|  | Total follow-up (year) | 82 166 | 50 431 |
|  | Mortality rate per 1000 person-year | 6.89 | 18.06 |
| **Respondents with missing data in one or more variables included in this study** | | | |
| **Men** | |  |  |
|  | Number population | 3 190 | 3 391 |
|  | Number of deaths | 386 | 1166 |
|  | Total follow-up (year) | 35 129 | 36 863 |
|  | Mortality rate per 1000 person-year | 10.99 | 31.63 |
| **Women** | |  |  |
|  | Number population | 4 181 | 4 816 |
|  | Number of deaths | 343 | 1142 |
|  | Total follow-up (year) | 46 846 | 53 923 |
|  | Mortality rate per 1000 person-year | 7.32 | 21.18 |
| **Incidence Rate Ratio of mortality comparing respondents with and without missing data** | | | |
| **Men** | | 1.03 (0.91-1.16) | 1.11 (1.03-1.19) |
| **Women** | | 1.06 (0.93-1.22) | 1.17 (1.07-1.28) |

Table S2 compares respondents with and without missing data in any of the variables included in the analysis. We estimated the incidence rate ratio of mortality comparing these two groups of respondents, to understand if respondents with missing data differ when compared with those without missing data.

**Table S3. Hazard ratio of mortality related to living alone among the Västerbotten population in 1990**

| **Variables** | | **Population aged 50+ who**  **lived in Västerbotten in 1990** | |
| --- | --- | --- | --- |
|  |  | **Men (n=38 046)** | **Women (n=43 232)** |
|  |  | **Hazard Ratio (95% Confidence Interval)** | **Hazard Ratio (95% Confidence Interval)** |
| **Family type and living arrangement** | | | |
|  | In partnership with children at home | Reference | Reference |
|  | In partnership without children at home | **1.05 (1.01-1.09)** | 1.03 (0.97-1.08) |
|  | Single parent with children at home | **1.13 (1.03-1.24)** | **1.17 (1.09-1.26)** |
|  | Single with no children at home | **1.32 (1.26-1.37)** | **1.20 (1.14-1.26)** |
| **Age group** | | | |
|  | 50-59 years | Reference | Reference |
|  | 60-69 years | **1.53 (1.47-1.59)** | **1.50 (1.43-1.57)** |
|  | 70-79 years | **2.77 (2.63-2.92)** | **2.47 (2.34-2.60)** |
|  | 80+ years | **5.44 (5.08-5.82)** | **5.72 (5.35-6.11)** |
| **Employment status** | | | |
|  | Fully employed | Reference | Reference |
|  | Intermittent employment | 1.05 (0.91-1.20) | 0.98 (0.86-1.13) |
|  | Mostly unemployed | 0.96 (0.78-1.19) | 1.09 (0.81-1.46) |
|  | Fully unemployed | **1.09 (1.00-1.18)** | **1.14 (1.05-1.24)** |
|  | Partly pensioner | **1.38 (1.31-1.45)** | **1.30 (1.22-1.38)** |
|  | Fully pensioner | **1.67 (1.61-1.75)** | **1.70 (1.62-1.78)** |
| **Education level** | | | |
|  | High education | Reference | Reference |
|  | Basic education | **1.24 (1.17-1.31)** | **1.33 (1.25-1.42)** |
|  | Middle education | **1.09 (1.03-1.16)** | **1.20 (1.12-1.29)** |
|  | Do not know | **2.03 (1.90-2.16)** | **2.32 (2.15-2.49)** |

Table S3 shows the hazard ratio of mortality based on living arrangement and controlled for age group, employment status and education level. This table corresponds to the results in Table 3, except that chronic disease risk factors and access to social capital were not controlled for in the analysis for Appendix 3. The data is presented as hazard ratio along with its 95% confidence interval. Only 95% CIs which have significance are bolded. All the data are presented for men and women separately.
